# A standardized extract of *Asparagus officinalis* stem prevents reduction in heat shock protein 70 expression in ultraviolet-B-irradiated normal human dermal fibroblasts: an in vitro study

**DOI:** 10.1186/s12199-018-0730-3

**Published:** 2018-08-21

**Authors:** Ken Shirato, Jun Takanari, Tomoko Koda, Takuya Sakurai, Junetsu Ogasawara, Hideki Ohno, Takako Kizaki

**Affiliations:** 1 Department of Molecular Predictive Medicine and Sport Science, Kyorin University School of Medicine, 6-20-2 Shinkawa, Mitaka, Tokyo, 181-8611 Japan; 2 Amino Up Chemical Co. Ltd, 363-32 Shin-ei, Kiyota, Sapporo, Hokkaido 004-0839 Japan; 3 Faculty of Nursing, Tokyo Healthcare University, 2-5-1 Higashigaoka, Meguro, Tokyo, 152-8558 Japan; 4 Department of Health Science, Asahikawa Medical University, 2-1-1-1 Midorigaoka-Higashi, Asahikawa, Hokkaido 078-8510 Japan; 5 Social Medical Corporation, The Yamatokai Foundation, 1-13-12 Nangai, Higashiyamato, Tokyo, 207-0014 Japan

**Keywords:** *Asparagus officinalis* L., Ultraviolet, Heat shock protein 70, Dermal fibroblasts, Photoaging, Skin health

## Abstract

**Background:**

Heat shock protein 70 (HSP70) exhibits protective effects against ultraviolet (UV)-induced premature skin aging. A standardized extract of *Asparagus officinalis* stem (EAS) is produced as a novel and unique functional food that induces HSP70 cellular expression. To elucidate the anti-photoaging potencies of EAS, we examined its effects on HSP70 expression levels in UV-B-irradiated normal human dermal fibroblasts (NHDFs).

**Methods:**

NHDFs were treated with 1 mg/mL of EAS or dextrin (vehicle control) prior to UV-B irradiation (20 mJ/cm^2^). After culturing NHDFs for different time periods, HSP70 mRNA and protein levels were analyzed using real-time polymerase chain reaction and western blotting, respectively.

**Results:**

UV-B-irradiated NHDFs showed reduced HSP70 mRNA levels after 1–6 h of culture, which were recovered after 24 h of culture. Treatment with EAS alone for 24 h increased HSP70 mRNA levels in the NHDFs, but the increase was not reflected in its protein levels. On the other hand, pretreatment with EAS abolished the UV-B irradiation-induced reduction in HSP70 expression at both mRNA and protein levels. These results suggest that EAS is capable to preserve HSP70 quantity in UV-B-irradiated NHDFs.

**Conclusions:**

EAS exhibits anti-photoaging potencies by preventing the reduction in HSP70 expression in UV-irradiated dermal fibroblasts.

**Electronic supplementary material:**

The online version of this article (10.1186/s12199-018-0730-3) contains supplementary material, which is available to authorized users.

## Background

The human skin is exposed to a variety of harmful environmental stressors, including ultraviolet (UV) radiation. UV rays produce reactive oxygen species in skin cells [1], which lead to DNA damage and result in cellular injury and dysfunction. Moreover, UV exposure induces pro-inflammatory responses in skin cells [2]. The resulting extracellular matrix breakdown accelerates photoaging.

Heat shock protein 70 (HSP70) holds non-native proteins generated by the stressors as soluble, folded intermediates in a refolding competent state, thereby exerting cytoprotective effects by preventing their aggregation [3]. HSP70 transgenic mice showed attenuated photoaging [4], suggesting that the preservation of the HSP70 contents in skin cells during UV exposure is beneficial in maintaining skin health.

A standardized extract of *Asparagus officinalis* stem (EAS) has been found to be a novel and unique functional food that induces the expression of HSP70 mRNA in peripheral leukocytes in an in vivo experiment [5]. Moreover, we recently reported that EAS suppressed the transactivation of pro-inflammatory mediators by inhibiting nuclear factor (NF-κB) signaling in adult-derived normal human dermal fibroblasts (NHDFs) exposed to UV-B irradiation [6].

However, it is not clear how HSP70 expression in NHDFs from adult donors responds to UV-B irradiation and whether EAS can modulate the cellular HSP70 expression. We hypothesized that EAS exerts anti-photoaging effects by increasing HSP70 expression in NHDFs. From the perspective of preventive medicine, it is meaningful to clarify the biological activities of EAS at a cellular level. Therefore, to further elucidate the anti-photoaging potencies of EAS, we examined its effects on the expression levels of HSP70 in UV-B-irradiated NHDFs.

## Methods

### Preparation of EAS

In the present study, the same batch of EAS (Amino Up Chemical Co. Ltd.) was used as in previous studies [6]. The procedure for preparing EAS is briefly described as follows [5, 7]: (1) fresh lower sections of the *Asparagus officinalis* stem were boiled in water at 121 °C for 45 min; (2) the boiled stem and the resulting extract were treated with cellulase and pectinase, both of which are widely used in the food industry; (3) after these enzymes were inactivated by incubation at 121 °C for 20 min, the extract was centrifuged and the resultant supernatant was mixed with dextrin (Pinedex; Matsutani Chemical Industry Co., Hyogo, Japan) as a filler; (4) the mixture was then concentrated in vacuo at 105 °C, sterilized at 121 °C for 45 min, and finally spray-dried to produce an EAS powder consisting of 52.6% EAS and 47.4% dextrin. In this study, the concentrations of EAS excluding dextrin are indicated.

### Cell culture

NHDFs from an adult donor (age, 47; sex, female; race, Caucasian; localization, temple; PromoCell, Heidelberg, Germany) were cultured in Fibroblast Growth Medium 2 (PromoCell). The cultures were maintained at 37 °C in a humidified incubator containing 5% CO_2_ in air. All the experiments were conducted using cells at passage 4. UV-B irradiation was administered using a UVM-57 Handheld UV Lamp (6-watt, 302 nm; UVP, LLC, Upland, CA, USA), from a distance of 7.5 cm from the cells. Irradiance was measured using a UV light meter (UV-340C; CUSTOM, Tokyo, Japan). To test the effect of UV-B, the cells were washed twice with phosphate-buffered saline and then irradiated with UV-B for 30 s (while they were under a thin layer of phosphate-buffered saline) to obtain a total dose of 20 mJ/cm^2^. To assess the effects of EAS, the cells were precultured with 1 mg/mL EAS or dextrin (vehicle control) for 3 or 24 h; immediately after UV-B irradiation, the cells were further cultured in the supplemented medium or under EAS-free condition for 3 h.

### Reverse transcription and real-time polymerase chain reaction (PCR)

Total cellular RNA was extracted using RNAiso Plus reagent (TaKaRa Bio, Shiga, Japan). One microgram of total cellular RNA was converted to single-stranded cDNA using the PrimeScript 1st Strand cDNA Synthesis Kit (Takara Bio). cDNA (1 μL) was amplified with the FastStart Universal Probe Master (Roche Life Science, Basel, Switzerland) using a 7500 Real-Time PCR System (Thermo Fisher Scientific, Waltham, MA, USA). The PCR conditions were 50 °C for 2 min and 95 °C for 15 s, followed by 45 cycles of 95 °C for 15 s and 60 °C for 1 min. The primer and fluorescent probe sequences were shown in Table 1. The HSP70 mRNA expression levels were calculated as the ratio of its value to that of the 18S rRNA (internal control).

**Table 1:** Primers and probes used in the real-time PCR

Protein	Gene	Probe	Forward primer sequence	Reverse primer sequence
HSP70	HSPA5	#64	5’-AGC TGT AGC GTA TGG TGC TG-3’	5′-AAG GGG ACA TAC ATC AAG CAG T-3’
18S rRNA	Rn18s	#55	5′-GGA GAA AAT CTG GCA CCA CAC CTT-3’	5’-CCT TAA TGT CAC GCA CGA TTT CCC-3’

### Western blotting

The denatured cellular proteins (10 μg) were separated via electrophoresis using a sodium dodecyl sulfate-polyacrylamide gel and then transferred to a polyvinylidene difluoride membrane. The primary antibodies against HSP70 or glyceraldehyde-3-phosphate dehydrogenase (GAPDH; Cell Signaling Technology, Danvers, MA, USA) were applied to the membranes at a 1/1000 dilution overnight. Next, the membranes were incubated with horseradish peroxidase-conjugated secondary antibodies (Jackson ImmunoResearch Laboratories, West Grove, PA, USA) at a 1/20,000 dilution for 30 min. After incubating with Clarity Western ECL Substrate (Bio-Rad Laboratories, Hercules, CA, USA), the membranes were exposed to X-ray films. The density of each protein band was quantified using the ImageJ software (National Institutes of Health, Bethesda, MD, USA). HSP70 protein expression levels were calculated as the ratio of its value to that of GAPDH (internal control).

### Telomere length

Genomic DNA was extracted using DNAiso reagent (TaKaRa Bio). Telomere length was analyzed by using the Absolute Human Telomere Length Quantification qPCR Assay Kit (ScienCell Research Laboratories, Sun Diego, CA). Genomic DNA (10 ng) was amplified with the FastStart Essential DNA Green Master (Roche Life Science) using a 7500 Real-Time PCR System (Thermo Fisher Scientific) and data analysis was conducted according to manufacturer’s instruction.

### Statistical analysis

The experimental data is presented as mean ± standard error of the mean (SEM). Differences between two groups were assessed using Student’s *t* test. Comparisons among at least three groups were carried out using one-way analysis of variance (ANOVA) and post hoc comparisons to determine the significant differences between the groups were performed using Tukey’s test. Differences were considered to be statistically significant when *p* < 0.05.

## Results and discussion

First, we analyzed the time course changes in the NHDF HSP70 mRNA levels after UV-B irradiation. The UV-B-irradiated NHDFs showed significant reduction in HSP70 mRNA levels after 1–6 h of culture, which were recovered to non-irradiated levels after 24 h of culture (Additional file 1: Figure S1). These results suggested that UV-B exposure impaired the cytoprotective effects of HSP70 by reducing its quantity in primary dermal fibroblasts. Previous studies showed that dermal fibroblasts isolated from a human neonate only expressed small amounts of the HSP70 protein under non-irradiated conditions, whereas UV-A and UV-B irradiation increased its quantity [8, 9]. This inconsistency in the effect of UV radiation on HSP70 expression in primary dermal fibroblasts may be explained by the difference in the age of the donors. It is possible that dermal fibroblasts from adult donors adapt to repeated exposure to environmental stressors during growth and increase the basal level of HSP70 expression and that UV-B irradiation transiently inhibits the acquired transcriptional induction and/or promotes HSP70 mRNA degradation. Therefore, preventing the reduction in HSP70 expression in UV-B-irradiated dermal fibroblasts can be crucial in maintaining skin health in adults. On the other hand, dermal fibroblasts used in this study are isolated from the skin in the temple area of adult female, although dermal fibroblasts from neonate are usually isolated from the foreskin after birth. Therefore, differences in sex and cell localization may also influence the responses to UV-B irradiation.

EAS, a standardized extract from the *Asparagus officinalis* stem, is a novel and unique functional food that induces HSP70 mRNA expression in peripheral leukocytes in an in vivo experiment [5]. In addition to the early finding, later studies demonstrated EAS treatment to increase HSP70 expression in HeLa cells and rat primary hepatocytes at both the mRNA and protein levels [7, 10]. Therefore, we further analyzed the effects of EAS treatment on HSP70 expression levels in NHDFs. As expected, EAS treatment increased HSP70 mRNA levels after 24 h of culture (Additional file 1: Figure S2A). However, the increase in HSP70 mRNA levels was not reflected in its protein levels (Additional file 1: Figure S2B). These null effects of EAS on HSP70 protein levels may be caused by its high basal levels in the dermal fibroblasts of adult donors.

**Fig. 1:**
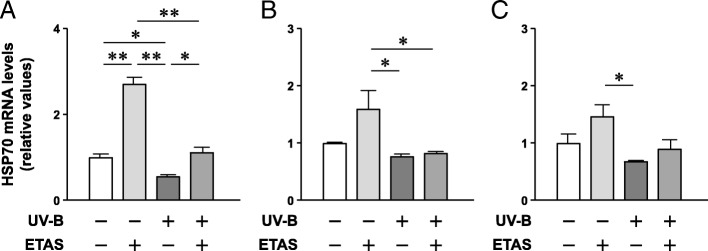
Effect of EAS pretreatment on the UV-B irradiation-induced reduction in HSP70 mRNA levels in NHDFs. The cells were treated with EAS or dextrin for 24 h (**a**) and 3 h (**b**); immediately after UV-B irradiation, the cells were further cultured in supplemented medium for 3 h. **c** Cells were treated with EAS or dextrin for 24 h; immediately after UV-B irradiation, the cells were further cultured under EAS-free condition for 3 h. The relative ratios of HSP70 with respect to 18S are shown. Mean ± SEM (*n* = 4). *p < 0.05; **p < 0.01 (one-way ANOVA and Tukey’s test)

Although EAS treatment alone did not influence the HSP70 protein levels in the NHDFs, we next examined if EAS prevented the UV-B irradiation-induced reduction in HSP70 expression. A 24-h EAS pretreatment and a subsequent treatment after UV-B irradiation completely recovered the UV-B irradiation-induced reduction in HSP70 mRNA levels (Fig. 1a). When the pretreatment period was shortened to 3 h, the recovery effect of EAS was almost abolished (Fig. 1b). Moreover, a 24-h EAS pretreatment without a subsequent treatment after UV-B irradiation demonstrated a propensity in recovering the reduced HSP70 mRNA levels, but this recovery was not significant (Fig. 1c). These results suggest that EAS contains active compounds that increase HSP70 mRNA production in the NHDFs, thereby preventing the reduction in HSP70 mRNA levels after UV-B irradiation and that it takes a sufficient duration of treatment with EAS before and after UV-B irradiation to obtain maximal effects.

**Fig. 2:**
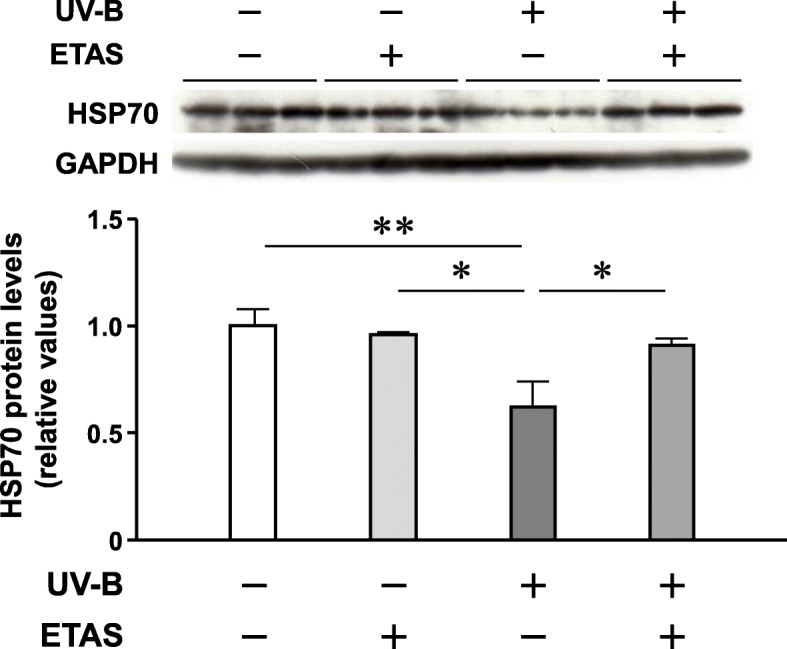
Effect of EAS pretreatment on the UV-B irradiation-induced reduction in HSP70 protein levels in NHDFs. The cells were treated with EAS or dextrin for 24 h; immediately after UV-B irradiation, the cells were further cultured in supplemented medium for 6 h. The relative ratios of HSP70 with respect to GAPDH are shown. Mean ± SEM (*n* = 3). *p < 0.05; **p < 0.01 (one-way ANOVA and Tukey’s test)

The preventive effects of EAS on the reduction in HSP70 expression in UV-B-irradiated NHDFs were also observed at the protein level (Fig. 2), which suggests that these effects protect the cells from UV-B damage. HSP70 is shown to exert anti-inflammatory effects by acting as a bridge among p65, ubiquitin E3 ligase, and proteasomes, thereby resulting in p65 degradation and suppression of NF-κB signaling [11]. We recently reported that EAS suppressed UV-B irradiation-induced interleukin-1β transactivation by inhibiting the NF-κB p65 nuclear translocation in the NHDFs [6]. However, it is unlikely that the anti-inflammatory behavior of EAS is mediated by the preservation of the intracellular HSP70 protein contents after UV-B irradiation, since the inhibitory effects of EAS on the p65 nuclear translocation can be observed in the 3-h subsequent treatment after UV-B irradiation, whereas the preventive effects on the reduction in HSP70 expression can only be observed after 24 h of pretreatment before UV-B irradiation.

Furthermore, HSP70 transgenic mice showed attenuated dermal fibroblast cell death after irradiation with UV-B [4], which suggests that the preservation of the intracellular HSP70 contents exerts a cytoprotective effect on the UV-B-induced cell death. Notably, EAS pretreatment did not prevent the reduction in the number of viable UV-B-irradiated NHDFs. It is conceivable that the complexity of EAS’s biological effects arises from multiple active compounds. Therefore, it is essential to identify these active compounds in order to elucidate the underlying mechanisms that determine the beneficial biological effects of EAS. Since it is well-established that HSP70 primarily influences protein folding by working as a molecular chaperone during non-native protein formation as in instances of UV exposure [3], EAS can be considered as a viable alternative in preventing the increase of non-native proteins and facilitating their aggregation in dermal fibroblasts.

Finally, to clarify whether EAS has the anti-aging effects as well as anti-photoaging effects, we analyzed the effects of UV-B and EAS on telomere length in NHDFs. However, absolute telomere length was not significantly changed by both UV-B irradiation and EAS treatment (Additional file 1: Figure S3). These results suggest that UV-B accelerates the machinery for photoaging but not aging of dermal fibroblasts and that EAS does not have a capability to modulate telomere length in dermal fibroblasts.

A study in progress by another group suggests that EAS contains proline-containing 3-alkyldiketopiperazine derivatives as HSP70 inducers. Oral EAS intake increased the mRNA levels of HSP70 in human peripheral leukocytes [5]. This previous finding suggests that the compounds or their metabolites directly act on the cells and induce HSP70 expression. Moreover, oral EAS intake attenuated sleep deprivation-induced stress responses, promoted good sleep, and increased salivary secretory immunoglobulin A levels in mice and humans [5, 7, 12]. Therefore, it is conceivable that the compounds or their metabolites are delivered to central nervous system after digestion ingestion via circulation. However, it may have a limitation, whether these active compounds are really delivered to peripheral skin tissues when EAS is orally taken. Although EAS clearly showed recovery effects on the reduction in HSP70 expression in UV-B-irradiated NHDFs in this in vitro study, further study is necessary to clarify whether its direct application as lotion or ointment is useful to maintain skin health.

As discussed above, although further studies are necessary for its practical application, EAS has been shown to have several beneficial biological actions against UV-B-irradiated NHDFs at a cellular level, including prevention of pro-inflammatory responses and HSP70 reduction. Recent [6] and present results will form a basis for future applied studies in an in vivo level. Therefore, from point of view of preventive medicine, EAS is a prospective material that has potentials to prevent photoaging, a skin pathological condition, induced by repeated UV ray exposure.

## Conclusion

The present findings demonstrate the potential of EAS to prevent photoaging by maintaining HSP70 contents in UV-B-irradiated dermal fibroblasts. Therefore, this readily available, inexpensive, and eco-friendly functional food may prove to be a useful component for dermatologic prophylactic strategies in maintaining skin health and function.

## Additional file

Additional file 1: **Figure S1.** The time course changes in NHDF HSP70 mRNA levels after UV-B irradiation. The cells were cultured for 1–24 h after UV-B irradiation. The relative ratios of HSP70 with respect to 18S rRNA are shown. Mean ± SEM (*n* = 3). *p < 0.05 (Student’s *t* test). **Figure S2**. Effect of EAS treatment on HSP70 expression levels in NHDFs. The cells were treated with EAS or dextrin for 24 h. HSP70 mRNA (A) and protein (B) levels were analyzed using real-time PCR and western blotting, respectively. The relative ratios of HSP70 with respect to 18S rRNA (A) and GAPDH (B) are shown. Mean ± SEM (*n* = 3). *p < 0.05 (Student’s *t* test). **Figure S3.** Effect of UV-B irradiation and EAS treatment on the absolute telomere length in NHDFs. The cells were treated with EAS or dextrin for 24 h; immediately after UV-B irradiation, the cells were further cultured in supplemented medium for 24 h. Mean ± SEM (*n* = 6). (PDF 139 kb)

## Abbreviations

ANOVA: Analysis of variance
GAPDH: Glyceraldehyde-3-phosphate dehydrogenase
HSP: Heat shock protein
NF-κB: Nuclear factor-κB
NHDFs: Normal human dermal fibroblasts
SEM: Standard error of the mean
UV: Ultraviolet
