# Supplementary material for: A standardized extract of Asparagus officinalis stem prevents reduction in heat shock protein 70 expression in ultraviolet-B-irradiated normal human dermal fibroblasts: an in vitro study
Source: Environ Health Prev Med. 2018 Aug 21;23:40. doi: 10.1186/s12199-018-0730-3 (PMC6104003; doi:10.1186/s12199-018-0730-3)
Supplement: Supplementary file 1 — Figure S1. The time course changes in NHDF HSP70 mRNA levels after UV-B irradiation. The cells were cultured for 1–24 h after UV-B irradiation. The relative ratios of HSP70 with respect to 18S rRNA are shown. Mean ± SEM (n = 3). *p < 0.05 (Student’s t test). Figure S2. Effect of EAS treatment on HSP70 expression levels in NHDFs. The cells were treated with EAS or dextrin for 24 h. HSP70 mRNA (A) and protein (B) levels were analyzed using real-time PCR and western blotting, respectively. The relative ratios of HSP70 with respect to 18S rRNA (A) and GAPDH (B) are shown. Mean ± SEM (n = 3). *p < 0.05 (Student’s t test). Figure S3. Effect of UV-B irradiation and EAS treatment on the absolute telomere length in NHDFs. The cells were treated with EAS or dextrin for 24 h; immediately after UV-B irradiation, the cells were further cultured in supplemented medium for 24 h. Mean ± SEM (n = 6). (PDF 139 kb) [file 12199_2018_730_MOESM1_ESM.pdf]

Environmental Health and Preventive Medicine

Short Communication

A standardized extract of *Asparagus officinalis* stem prevents reduction in heat shock protein 70 expression in ultraviolet-B-irradiated normal human dermal fibroblasts: an *in vitro* study

Ken Shirato[1*], Jun Takanari[2], Tomoko Koda[3], Takuya Sakurai[1], Junetsu Ogasawara[4], Hideki Ohno[5] and Takako Kizaki[1]

[1]Department of Molecular Predictive Medicine and Sport Science, Kyorin University School of Medicine, 6-20-2 Shinkawa, Mitaka, Tokyo 181-8611, Japan

[2]Amino Up Chemical Co. Ltd., 363-32 Shin-ei, Kiyota, Sapporo, Hokkaido 004-0839, Japan

[3]Faculty of Nursing, Tokyo Healthcare University, 2-5-1 Higashigaoka, Meguro, Tokyo 152-8558, Japan

[4]Department of Health Science, Asahikawa Medical University, 2-1-1-1 Midorigaoka-Higashi, Asahikawa, Hokkaido 078-8510, Japan

[5]Social Medical Corporation, the Yamatokai Foundation, 1-13-12 Nangai, Higashiyamato, Tokyo 207-0014, Japan

E-mail address: shirato@ks.kyorin-u.ac.jp (K. Shirato); takanari@aminoup.co.jp (J. Takanari); t-kouda@thcu.ac.jp (T. Koda); ohnoh2o@yamatokai.or.jp (H. Ohno); kizaki@ks.kyorin-u.ac.jp (T. Kizaki)

* Corresponding author: shirato@ks.kyorin-u.ac.jp

[1]Department of Molecular Predictive Medicine and Sport Science, Kyorin University School of Medicine, 6-20-2 Shinkawa, Mitaka, Tokyo 181-8611, Japan

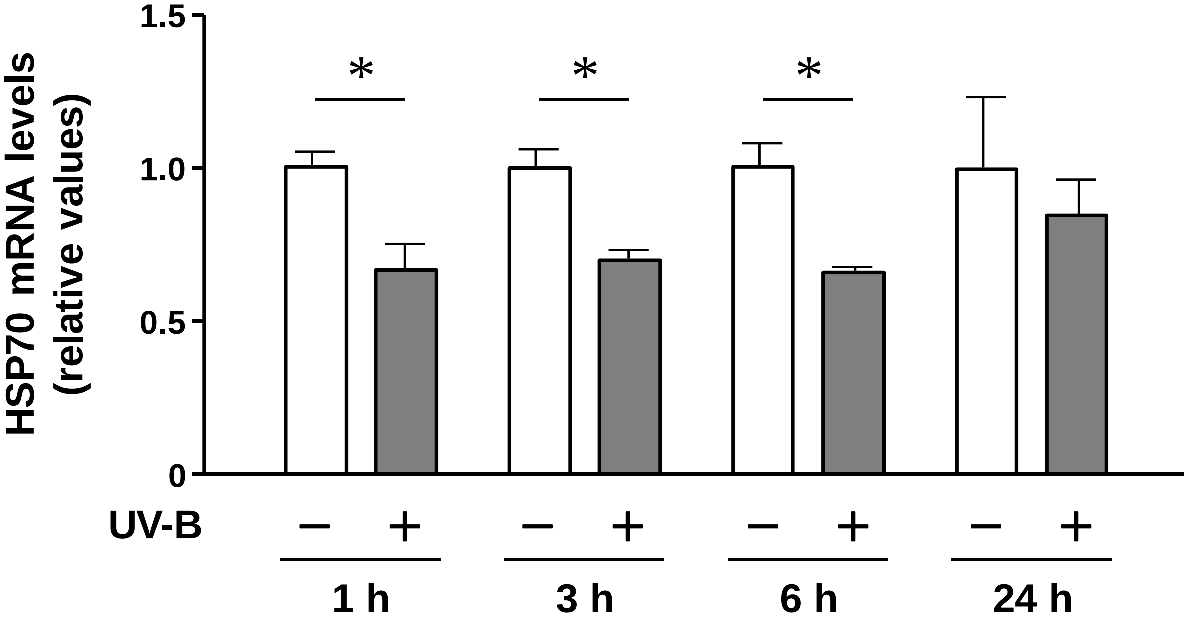

Fig. S1 The time-course changes in NHDF HSP70 mRNA levels after UV-B irradiation. The cells were cultured for 1–24 h after UV-B irradiation. The relative ratios of HSP70 with respect to 18S rRNA are shown. Mean ± SEM (n = 3). *$p < 0.05$ (Student's t-test).

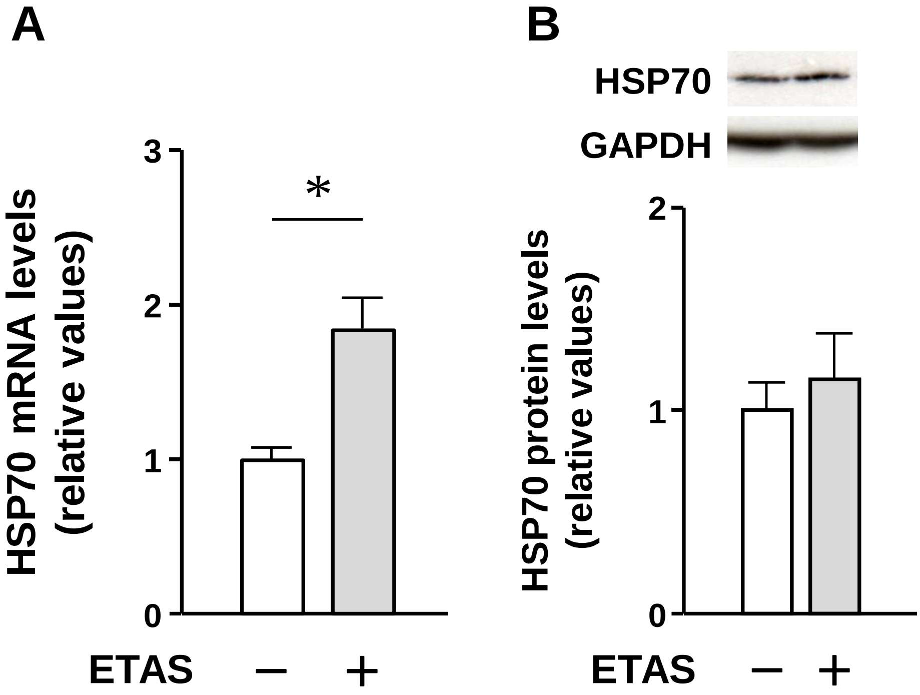

Fig. S2 Effect of EAS treatment on HSP70 expression levels in NHDFs. The cells were treated with EAS or dextrin for 24 h. HSP70 mRNA (A) and protein (B) levels were analyzed using real-time PCR and western blotting, respectively. The relative ratios of HSP70 with respect to 18S rRNA (A) and GAPDH (B) are shown. Mean ± SEM (n = 3). *$p < 0.05$ (Student's t-test).

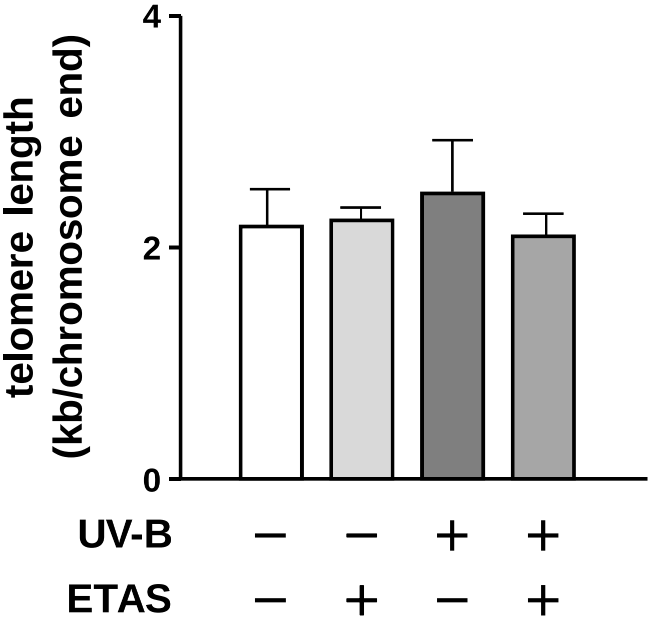

Fig. S3 Effect of UV-B irradiation and EAS treatment on the absolute telomere length in NHDFs. The cells were treated with EAS or dextrin for 24 h; immediately after UV-B irradiation, the cells were further cultured in supplemented medium for 24 h. Mean ± SEM (n = 6).
